# SeUneter: Channel attentive U-Net for instance segmentation of the cervical spine MRI medical image

**DOI:** 10.3389/fphys.2022.1081441

**Published:** 2022-12-06

**Authors:** Xiang Zhang, Yi Yang, Yi-Wei Shen, Ping Li, Yuan Zhong, Jing Zhou, Ke-Rui Zhang, Chang-Yong Shen, Yi Li, Meng-Fei Zhang, Long-Hai Pan, Li-Tai Ma, Hao Liu

**Affiliations:** ^1^ Department of Orthopedics, Orthopedic Research Institute, West China Hospital, Sichuan University, Chengdu, China; ^2^ School of Computer Science, Southwest Petroleum University, Chengdu, China; ^3^ West China School of Medicine, Sichuan University, Chengdu, China

**Keywords:** MRI image segmentation, U-Net, data augmentation, channel attention, cervical spine

## Abstract

In recent years, cervical spondylosis has become one of the most common chronic diseases and has received much attention from the public. Magnetic resonance imaging (MRI) is the most widely used imaging modality for the diagnosis of degenerative cervical spondylosis. The manual identification and segmentation of the cervical spine on MRI makes it a laborious, time-consuming, and error-prone process. In this work, we collected a new dataset of 300 patients with a total of 600 cervical spine images in the MRI T2-weighted (T2W) modality for the first time, which included the cervical spine, intervertebral discs, spinal cord, and spinal canal information. A new instance segmentation approach called SeUneter was proposed for cervical spine segmentation. SeUneter expanded the depth of the network structure based on the original U-Net and added a channel attention module to the double convolution of the feature extraction. SeUneter could enhance the semantic information of the segmentation and weaken the characteristic information of non-segmentation to the screen for important feature channels in double convolution. In the meantime, to alleviate the over-fitting of the model under insufficient samples, the Cutout was used to crop the pixel information in the original image at random positions of a fixed size, and the number of training samples in the original data was increased. Prior knowledge of the data was used to optimize the segmentation results by a post-process to improve the segmentation performance. The mean of Intersection Over Union (mIOU) was calculated for the different categories, while the mean of the Dice similarity coefficient (mDSC) and mIOU were calculated to compare the segmentation results of different deep learning models for all categories. Compared with multiple models under the same experimental settings, our proposed SeUneter’s performance was superior to U-Net, AttU-Net, UNet++, DeepLab-v3+, TransUNet, and Swin-Unet on the spinal cord with mIOU of 86.34% and the spinal canal with mIOU of 73.44%. The SeUneter matched or exceeded the performance of the aforementioned segmentation models when segmenting vertebral bodies or intervertebral discs. Among all models, SeUneter achieved the highest mIOU and mDSC of 82.73% and 90.66%, respectively, for the whole cervical spine.

## 1 Introduction

Degenerative cervical spondylosis is a chronic and progressive degeneration of osseocartilaginous components of the cervical spine that is usually related to wear and tear during aging (Theodore, 2020). Patients with degenerative cervical spondylosis may present with neck pain, cervical radiculopathy, or myelopathy due to the compression and inflammation of the nerve roots and spinal cord (Voorhies, 2001). A recent Global Burden of Disease study estimated that nearly a third of a billion people worldwide had neck pain (Dieleman et al., 2020; Safiri et al., 2020). Despite the huge personal and socioeconomic burden that neck pain causes, it receives only a fraction of research attention and publicity compared to low back pain (Cohen, 2015). Medical imaging techniques such as radiographs, computed tomography (CT), and magnetic resonance imaging (MRI) are widely used in the diagnosis of degenerative cervical spondylosis. Of them, MRI is the most used imaging modality for the diagnosis of the cervical degenerative disc disease and neurological compression because it can clearly show the anatomical details of the cervical spine, including the vertebral body, intervertebral disc, spinal canal, and spinal cord. However, in current clinical practice, the manual identification and segmentation of the components of the cervical spine on MRI make it a laborious, time-consuming, and error-prone process, particularly in basic medical institutions. The development of an MRI-based automated and accurate segmentation system of cervical spine components is urgently needed. Several kinds of literature reported the automated segmentation of the lumbar spine based on deep learning.

In recent years, artificial intelligence technology has played an important role in medical imaging processing. Convolutional neural networks (CNN) have unique advantages in image processing with their special structure of local weight sharing and are widely used in various downstream tasks. In semantic segmentation, the common architectural design was mainly the encoder–decoder structure, such as FCN (Shelhamer et al., 2017), U-Net, and DeepLab-v3+ (Chen et al., 2018). Among them, the structure of U-Net could often achieve superior performance when the number of samples was insufficient, so various U-Net-based methods were proposed. H-DenseUNet (Li et al., 2018) included a 2D DenseUNet for efficiently extracting intra-slice features and a 3D DenseUNet aggregation for the liver and tumor segmentation. UNet++ (Zhou et al., 2018) added the nested and dense skipped connections based on U-Net. The nnU-Net (Isensee et al., 2019) was a robust adaptive framework based on 2D and 3D U-Nets. The subsequently proposed UNet3+ (Huang et al., 2020) used full-scale skipped connections to fuse feature maps of different scales for segmenting the positions and boundaries of organs in images. A novel Low-cost U-Net (LCU-Net) (Zhang et al., 2021a) for the environmental microorganism (EM) image segmentation task was proposed to assist microbiologists in detecting and identifying EMs more effectively. In addition, the attention mechanism was also introduced into segmentation tasks in medical image segmentation. For example, attention U-Net (Oktay et al., 2018) was integrated based on the U-Net and attention gates. Several researchers had introduced the transformer (Vaswani et al., 2017) to computer vision tasks. Some models added transformer modules or completely relied on the transformer to design segmentation networks. U-Net (Hatamizadeh et al., 2021) applied the transformer as an encoder to learn a sequential representation of the input volume and capture global multi-scale information efficiently, while also following the design of a successful “U-shaped” network. TransUNet (Chen et al., 2021) took the transformer as the basic network architecture and combined it with U-Net to enhance the finer details by recovering the local spatial information of the image. It was an alternative framework for the main medical image segmentation methods based on FCN. Swin-Unet (Cao et al., 2021) was the first pure transformer-based U-shaped architecture, using the hierarchical Swin Transformer (Liu et al., 2021) with shifted windows as the encoder to extract contextual features.

Deep learning (DL) algorithms were also widely used in image segmentation of MRI datasets. In 2021, a DL-based lumbar spine MRI segmentation method was proposed (Li et al., 2021). At the same time, a new detection-guided hybrid supervised segmentation network (Suri et al., 2021) was proposed to achieve automatic lumbar spine segmentation on T2-weighted (T2W) MRI. The newly proposed DGMSNet (Pang et al., 2022) network enabled the automated, multimodal segmentation of vertebral bodies and intervertebral discs, while the concurrently proposed 2D U-Net model (Hwang et al., 2021) segmented the lumbar bone marrow in sagittal T1-weighted MRI. Zhang and Wang (2021) proposed a novel segmentation method for the cervical vertebrae based on PointNet++ and converging segmentation. For spinal cord segmentation, some methods based on the morphology, region, and watershed were used to judge and segment spinal cord information (Ahammad and Rajesh, 2018; Garg and Bhagyashree, 2021).

However, the current segmentation tasks mainly involved the segmentation tasks of the lumbar and thoracic spine, and the research on the segmentation of the cervical spine MRI images was still lacking. Given the smaller vertebral bodies and discs compared to the lumbar spine, the greater variation in endplate shape and the lack of consideration of the spinal cord data and the relative segmentation task of the cervical spine remained a challenge. Many current studies only focused on the segmentation of the cervical vertebral mass or the segmentation of the spinal cord, without joint-training segmentation, or fail to achieve the end-to-end goal by multi-step segmentation. The identification of the cervical spinal cord together with the spinal canal was of critical importance for further automated diagnosis of spinal stenosis or neural compression. Therefore, we aimed to develop a new segmentation method for the automatic segmentation of the cervical spine MRI image, including the cervical vertebrae, cervical disc, spinal cord, and spinal canal. Considering that the majority of cervical spine disorders were diagnosed by MRI, our proposed model held great promise for the automatic diagnosis of cervical spine disorders.

In this paper, a new dataset of cervical spine MRI was collected. The images were acquired in the T2-weighted (T2W) mode, including the cervical vertebral body, intervertebral disc, spinal canal, and spinal cord. The whole MRI dataset contained 600 images in total, with 16 categories (including the background), which was used for instance segmentation in this work.

To make the dataset more suitable for the segmentation task and alleviate the problem of insufficient samples, we proposed a new improved model termed SeUneter based on the classical U-Net architecture (Ronneberger et al., 2015), which enjoyed the main structure of U-Net, including skipped connections and a U-shaped structure. SeUneter adjusted the network depth and introduced the channel attention mechanism into U-Net. Specifically, through deepening the depth of the network and improving its extraction of feature information, the down-sampling process was extended to a depth of five layers, and its dimension changes to 1024 × 16×16. In addition, in order to expand the feature extraction capability of the double-layer convolution (double conv) in U-Net, a channel attention module (SeNet (Hu et al., 2018)) was added to the double convolution in each layer, in order to learn the information of channels in the convolution process. This way, it could enhance the channels that are beneficial to the segmentation results and weaken the adverse effects, to improve the segmentation ability of the model. Moreover, we leveraged the characteristics of the dataset to optimize and adjust the segmentation results through prior knowledge. On the basis of model adjustment, we used a data augmentation strategy named Cutout (Devries and Taylor, 2017) to increase the availability of the data samples and alleviate over-fitting.

Our contributions could be summarized as follows: 1) the SeUneter method was proposed to improve the performance of the original U-Net. SeUneter deepened the network layer at the network level to achieve the extraction of deep characteristics by adding channel attention in the original dual-layer convolution and transferring the characteristics in the channel to the direction that was conducive to image segmentation. 2) Enhancing the training data through data augmentation Cutout eased the over-fitting situation to a certain extent and achieved segmentation improvement. 3) The structure of the segmentation result and the interior of the segmentation were adjusted and optimized according to the prior knowledge of the cervical spine MRI medical image data. 4) A medical image MRI dataset for cervical spine segmentation at our institution was collected, including 600 MRI images of 300 patients, each image contains 15 segmentation class labels and background (sixteen classes in total), which could be used for instance segmentation.

## 2 Methods and materials

### 2.1 Dataset

This study was approved by the Ethics Committee of Biomedical Research, West China Hospital of Sichuan University. Written informed consent was waived because of the retrospective nature of data collection (age/gender) and the use of de-identified MRI images. A total of 319 consecutive patients in an age range between 18 and 95, who were prescribed cervical MRI for medical reasons and who were scanned between 2019 and 2021 using either of the Siemens 3.0-T scanners at the West China Hospital of Sichuan University. In all, 19 patients were excluded for the following reasons: 1) incomplete image of the cervical spine (*n* = 17); 2) insufficient MRI quality (*n* = 3). Finally, 300 patients were retrospectively collected in this study.

The images were acquired in the T2-weighted (T2W), including the cervical vertebral body, intervertebral disc, spinal canal, and spinal cord. The whole MRI dataset contained 600 images in total, with 16 categories: cervical bodies: C2, C3, C4, C5, C6, C7, and T1; cervical intervertebral disc: C2/3, C3/4, C4/5, C5/6, C6/7, and C7/T1; spinal canal; spinal cord; and background. Since each MRI contained a complete set of 16 categories, the total dataset had a total of 600 of each category.

### 2.2 Approach

The slices of MRI images were thicker than other medical images such as CT; hence, there were relatively fewer available MRI images. However, the insufficient volume of data may cause the models to over-fit during the training process. The U-shaped structure could concatenate the same-level information of an encoder and decoder into a small number of medical samples, thus providing more refined features for segmentation, alleviating over-fitting, and improving the segmentation ability to a certain extent.

**FIGURE 1:**
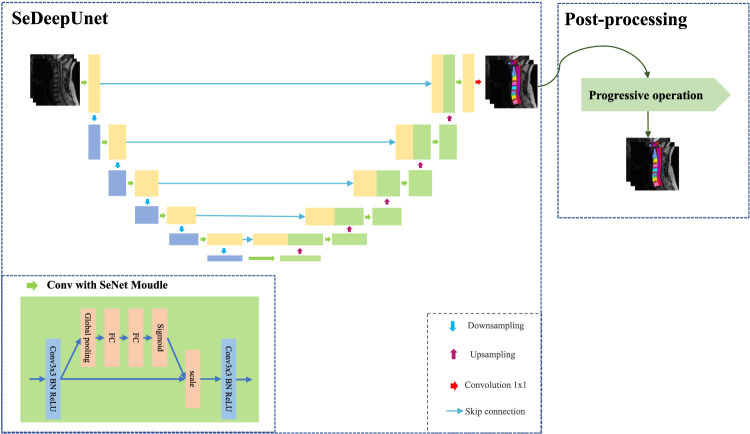
Diagram of the SeUneter approach.

In this paper, considering the size information of MRI images and the use of the channel attention mechanism in the convolutional network, we propose an improved U-Net method, called SeUneter, which contains two components: the SeDeepUnet model and post-processing. SeDeepUnet realizes the segmentation of the cervical spine MRI image, and post-processing is used to adjust the segmentation results by using prior knowledge about cervical spine MRI images to obtain more precise results. As shown in Figure 1, the overall structure design of the SeDeepUnet model is like the U-Net structure, while it deepens the layers and further samples to the 16 × 16 size to extract more detailed feature information and designs the different channel weight during the further convolutional process formed through the convolution. Furthermore, the double conv is re-designed by adding the SeNet channel attention module. In the meantime, to reduce the over-fitting phenomenon brought on by less data, the original training data have been augmented by Cutout.

#### 2.2.1 Data augmentation

Deep neural networks often over-fit when it learns from less data, which is due to the difficulty of capturing features and requires a more complicated network design. Hence, the low data volume is not enough to support the training of models. Then, increasing the volume of training data samples through a certain data augmentation method can alleviate the excess of the model to a certain extent. Considering that the photos of the MRI cervical spine medical images (such as the shooting posture, and the position of the cervical spine) are similar on the whole, the division of the MRI cervical spine images is a fine-grained learning problem. Therefore, the Cutout method for data augmentation of MRI images is adopted in our approach, to improve the robustness and overall performance of convolutional neural networks.

The MRI dataset was divided into training and testing parts, and the training concentration includes 175 cases; each case only contained two more clear MRI cervical spine images. To retain the original details of the original image, the image was not cropped or resized and the size was maintained at 512 × 512, but the process of data augmentation by the Cutout method (Devries and Taylor, 2017) was performed, which expanded the number to 700 samples.

The Cutout contained two parameters: the number of masks ${Hole}_{n}$ and the hole border that needed to be masked ${Mask}_{l}$ . The Cutout erased the original image information by randomly selecting a square area of a fixed size and then using an all-0 fill method. In this work, we set the ${Hole}_{n}$ as 5 and the ${Mask}_{l}$ as 47, which were cut randomly for each picture. Masking the local information so that most feature information is retained, the generalization ability of the model is improved.

$$mask\left({rand}_{x}:{rand}_{x}+{Mask}_{l},{rand}_{y}:{rand}_{y}+{Mask}_{l}\right)=0.$$ (1)

image=image ∗ mask. (2)

The mask was constructed to erase the original sample, ${rand}_{x}$ was a random value based on the image width, ${rand}_{y}$ was a random value based on the image height, ${Mask}_{l}$ was the edge length of the square filled with zero, image was the original image, and additionally, ${Hole}_{n}$ was the number of squares to be erased.

**FIGURE 2:**
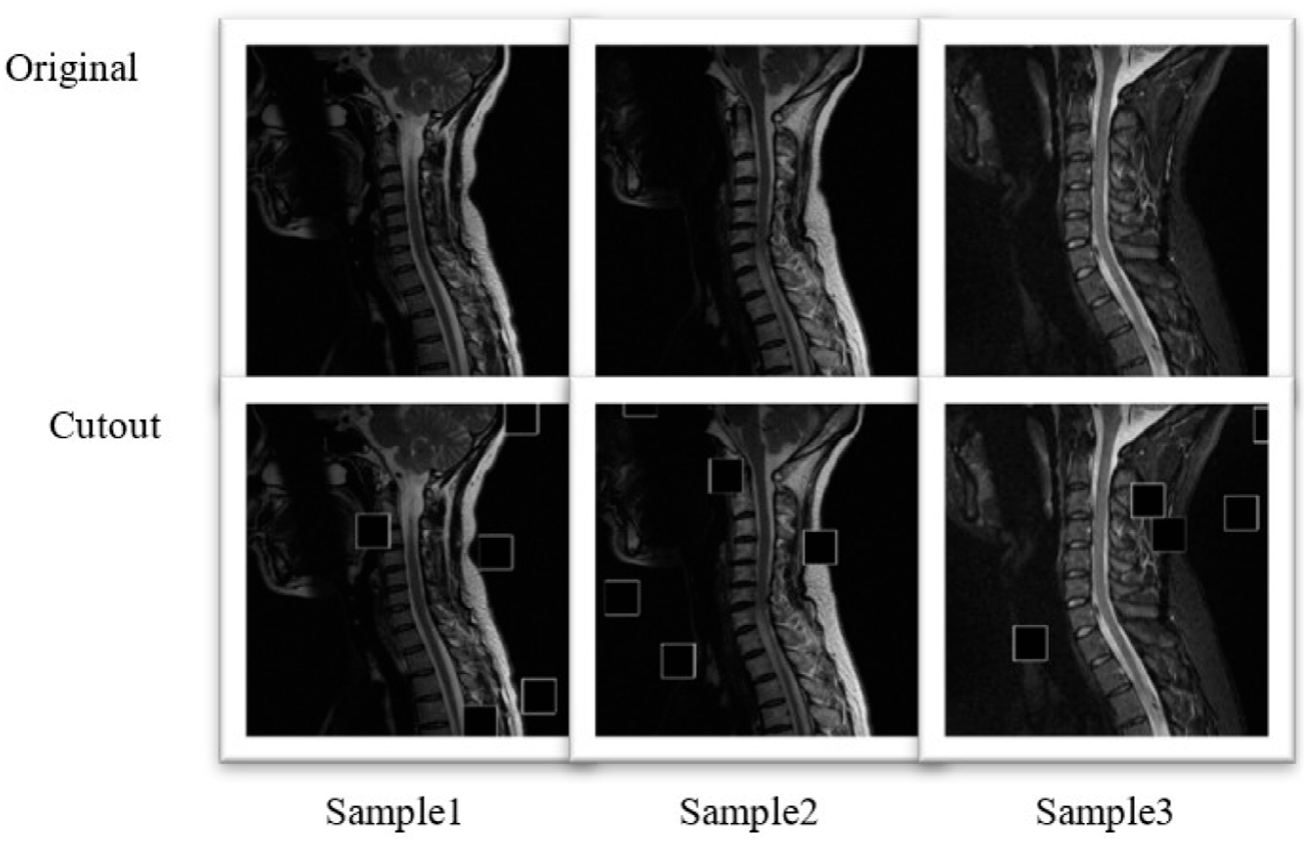
Comparison image of the original sample and the augmented sample of the three diagrams.

The comparison image of the original sample and the augmented sample is shown in Figure 2. A small square area was randomly selected for an image, and the pixel value in this area was set to 0. The Cutout may enable CNNs to make better use of the global information about an image, rather than relying on a small set of specific visual features.

#### 2.2.2 The SeDeepUnet model

As Figure 1 shows, for extracting more underlying image information, the SeDeepUnet’s structure grows to a deeper dimension. The encoding part contains five lower sampling modules, and the number of channels is increased to 1,024 to extract features of the cervical spine data. The decoding part contains five upper sampling modules, which are used to restore the image feature extraction to the original image size and implement the pixel segmentation.

In addition to deepening the network layer, considering that double conv operations in traditional U-Net did not analyze the importance of the channel, the channels after equal convolution are defective. The effects of different channels will have different effects, so the effect of individual channels is distributed by adding a channel attention mechanism in the current network layer.

The adverse channel is weakened, and the benefit is enhanced; the SeNet module can extract more useful feature information. Specifically, SeNet first uses the squeeze operation of the feature map obtained by convolution to obtain the global characteristics of the channel level and then uses the excitation operation of the global features to learn the relationship between each channel. In essence, SeNet is an attention mechanism or door control operation in the channel dimension. As shown in Figure 1, SeNet is applied to each double conv of U-Net to realize the attention of the channel, and it contains more information for the important channel, to reduce the impact of interference.

#### 2.2.3 Model learning

In our model, the objective is the combined classification loss (i.e., cross-entropy) and Dice Loss. The cross-entropy loss (CeLoss) evaluates the class prediction of each pixel vector individually and then averages over all pixels, which gives a better prediction of the global information of the image. The Dice Loss is used to evaluate the similarity of two sample statistics, which essentially measures the overlap of the two samples. The Dice Loss (DeLoss) is equivalent to global examination, while cross-entropy is approximated pixel-by-pixel microscopically, with complementary perspectives, which can alleviate the situation when there is an extreme imbalance between the front and back view and when there is an imbalance in the segmentation content, while CeLoss can play a guiding role for Dice Loss. We use the combination of the cross-entropy function, CeLoss1, and the DeLoss2 function to show the degree of difference between the prediction and the actual data, as shown in Eqs 3, 4, 5:

$$CeLoss=-\frac{1}{N}\overset{N}{\underset{i=1}{\sum }}\sum_{c=1}^{C}g_{i}^{c}\log s_{i}^{c},$$ (3)

$$DeLoss=1-\frac{2\left|y⋂\hat{y}\right|}{\left|y\right|+\left|\hat{y}\right|},$$ (4)

$$Loss=\lambda CeLoss+\left(1-\lambda \right)DeLoss.$$ (5)

Cross-entropy (CE) was a measure of the difference between two distributions, where i was each pixel, c is the classification, $g_{i}^{c}$ was an indication of whether the classification was correct, and $s_{i}^{c}$ was the probability of being classified into a certain class. Dice was used to calculate the similarity between two images, where y represented the true segmentation label and $\hat{y}$ represented the segmentation result predicted by the model. The loss function allocated the cross-entropy loss function and the dice loss function with λ. We set λ to 0.5 in the experiment.

#### 2.2.4 Post-processing

**FIGURE 3:**
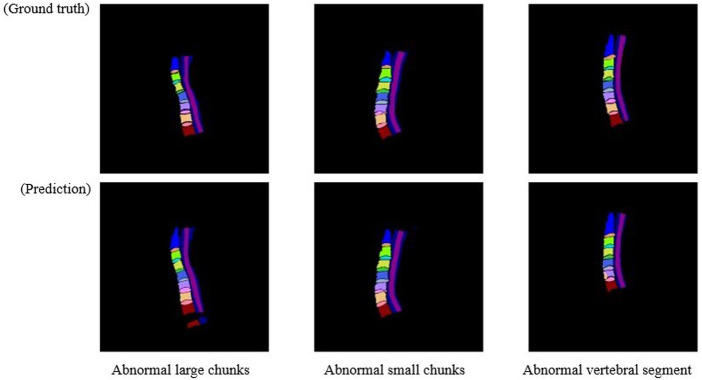
Three main anomaly segmentation cases from the validation set.

**FIGURE 4:**
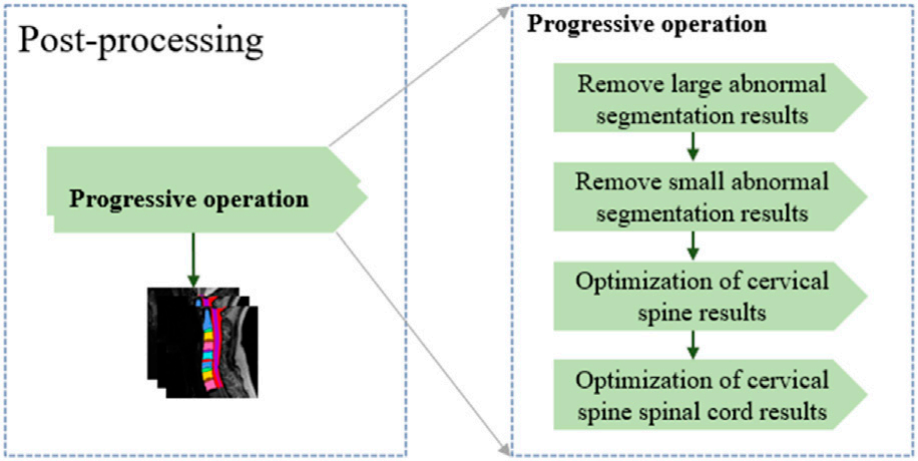
Multiple progressive modules for the post-processing section.

In general, after training using the training dataset, deep learning models are directly used to perform the segmentation. However, there are some cases that are not expected. As shown in Figure 3, three main abnormal segmentation cases are present in the validation set. In the proposed method, fine-tuning is performed according to the segmentation results and the relevant knowledge of the data itself after the segmentation task to overcome the aforementioned abnormal cases. As shown in Figure 4, the post-processing of the current cervical spine data is mainly divided into two parts: the first two are external optimizations and the next two are internal optimizations.

##### 2.2.4.1 Optimizing external segmentation results

Considering that the number of labeling classes in the current cervical spine data is consistent, we first remove the large block anomaly segmentation. For the large redundant anomaly, segmentation result Y_P_, its structure is often very different from that of the labeled Y_L_. Therefore, a larger size kernel, Ker_L_, is used to perform the connected domain operation, and the obtained block information $Y_{\frac{1}{B}}$ is used to judge the structural abnormality of the block segmentation result in Y_P_. A larger threshold η_l_ is used to filter the block information. If there is a large abnormal structure, after comparing with a large threshold, the segmentation results below the threshold will be replaced and modified into background labels; otherwise, the category information of the original segmentation results will be retained.

In contrast, after the removal of the larger abnormal segmentation results is completed, some small block abnormal segmentation information is still hidden in the segmentation results. At this time, through the connected domain operation with the small kernel size, the segmentation results after the first optimization are classified into fine-grained. Considering that the single cervical vertebra segmentation has a certain size, a smaller threshold η_s_ is set, and the pixel category below the current threshold information is classified as the background label.

##### 2.2.4.2 Optimizing internal segmentation results

After completing the two-step abnormal optimization of the external segmentation result, the abnormal structure in the segmentation result is eliminated. The adjustment basis for the internal segmentation result comes from the unified correction of the segmentation result and the connected domain. Considering that there may be a large connection relationship between the spinal cord information and multiple cervical vertebrae during segmentation, the original segmentation results are separated according to the spinal cord and cervical vertebrae.

If the number of segmentation classes that remove the spinal cord information (class = 14) is equal to the result of the connected domain, it is considered that the connected domain brings segmentation information, which is equivalent to the segmentation result, and the segmentation result is perfected according to the result of the connected domain. If the number of results of the connected domain segmented is greater than the current number of segmentation classes, it is considered that the segmentation result contains additional abnormal information. The segmentation result is adjusted to hide the additional information about the current connected domain and adjust the result.

The fact that the connected domain segmentation result is smaller than the number of classes is mainly because of the adhesion in the vertebral disc of the segmentation result. Therefore, the cervical spine and the intervertebral disc are separated. Considering the small volume of the intervertebral disc, only the original segmentation was adjusted according to its segmentation result. For the segmentation of the intervertebral disc, considering its adhesion with the spinal cord information, an additional number of categories will be introduced under the connected domain operation. However, this part of the additional information is relatively small, so a smaller new threshold is introduced here to eliminate the influence of spinal cord information and then performing the segmentation according to the connected domain, adjusting the original segmentation result. In contrast, the adjustment of the segmentation results of the spinal cord and other information only fills the inner cavity, which has little effect on the change of the results.

### 2.3 Experiment

#### 2.3.1 Data description

**FIGURE 5:**
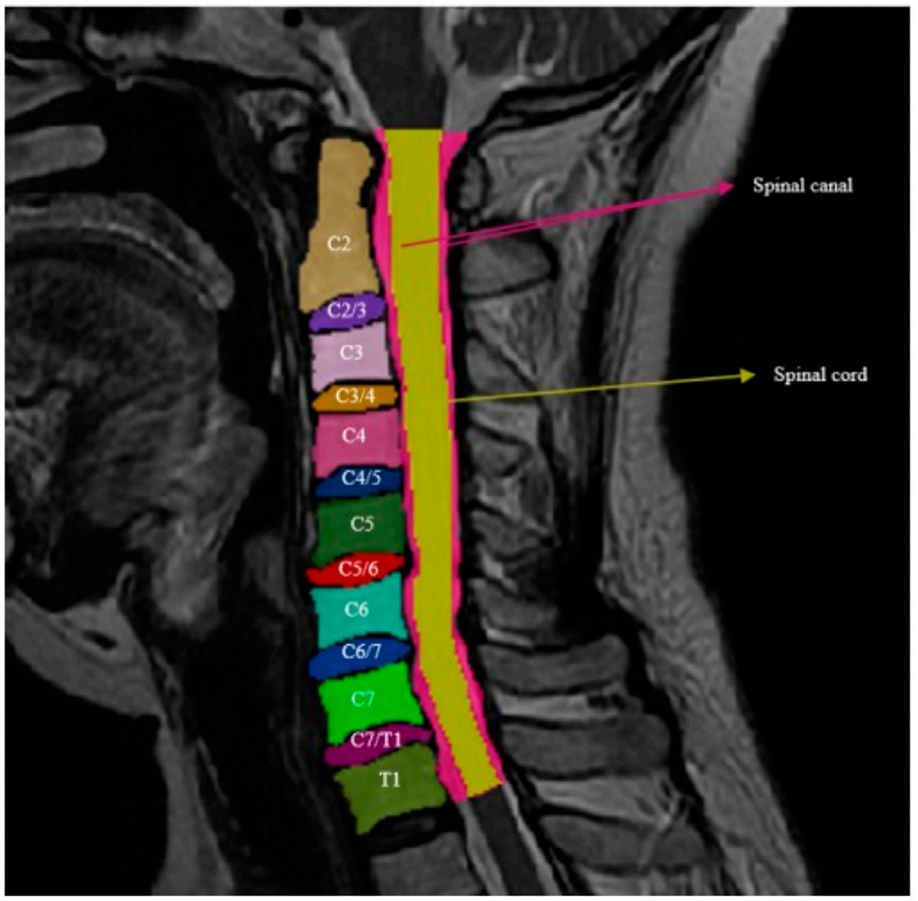
Cervical bodies: C2, C3, C4, C5, C6, C7, and T1; cervical intervertebral disc: C2/3, C3/4, C4/5, C5/6, C6/7, and C7/T1; spinal canal; and spine cord.

This experiment uses 2D image data in the T2-weighted (T2W) mode of MRI, where each patient is labeled with two slices. These annotated data were converted into the png format needed for the model. The classes to be segmented are 16 (including the background) (Figure 5).

#### 2.3.2 Experiment setting

In the experimental training process, we set the relevant parameters of the training process, the learning rate of the model is uniformly set to 1e-05, batch size is 6, epoch is 200, the backpropagation method is RMSprop, and the measured metric is the mean Dice similarity coefficient. The U-Net, AttU-Net (Attention U-Net), UNet ++, DeepLab-v3+, Swin-Unet, and TransUNet are selected for experimental comparison. Measures of interest include the mean of IOU (mIOU) index for the different categories and the mean of Dice similarity coefficient (mDSC) and mIOU for all classes. A 10-time validation strategy was performed on each model. Among the 600 images, 350 images are used for training sets, 100 validation sets, and 150 test sets.

### 2.4 Statistical analysis

The Shapiro–Wilk test verified the normality of the data distribution, and the data that satisfied a normal distribution are represented by the mean value and standard deviation. A paired *t*-test was adopted for comparisons between our method and baseline methods (the U-Net, AttU-Net (Attention U-Net), UNet ++, DeepLab-v3+, Swin-Unet, and TransUNet). *p* < 0.05 was considered statistically significant.

## 3 Results and discussions

### 3.1 Ablation experiment

For different adjustments of the model, the effects of different modules on the results are verified according to their progressive relationship, and the results brought by different adjustments are shown in Table 1. Among them, A1 means adding the SeNet module, A2 increases the model depth in the previous process, A3 adds data enhancement operations, and A4 introduces post-processing operations in the segmentation results to optimize the segmentation performance. When adding the module of A1, the U-Net + A1 performed significantly better in terms of the mDSC compared to U-Net (87.24 ± 1.70 *vs.* 85.18 ± 1.61; *p* = 0.009). In addition, the mDSC was improved by more than 5% using A1+A2+A3+A4 (90.67 ± 1.63 *vs.* 85.18 ± 1.61; *p* < 0.05). The U-Net combined with A2, A3, and A4 performed significantly better in terms of the mDSC compared to U-Net + A1 (90.67 ± 1.63 *vs.* 87.24 ± 1.70; *p* < 0.001).

**TABLE 1:** Performance improvement of the model after the stacking of different modules (%).

Metrics	U-Net	U-Net + A1	U-Net + A1 + A2	U-Net + A1 + A2 + A3	U-Net + A1 + A2 + A3 + A4
DSC (±STD)	85.18 ± 1.61	87.24 ± 1.70	89.00 ± 1.66	90.14 ± 1.62	90.67 ± 1.63

Note: DSC, Dice similarity coefficient; STD, standard deviation; and A1 means adding the SeNet module, A2 increases the model depth in the previous process, A3 adds data enhancement operations, and A4 introduces post-processing operations in the segmentation results to optimize the segmentation performance.

### 3.2 Image augmentation comparison

**FIGURE 6:**
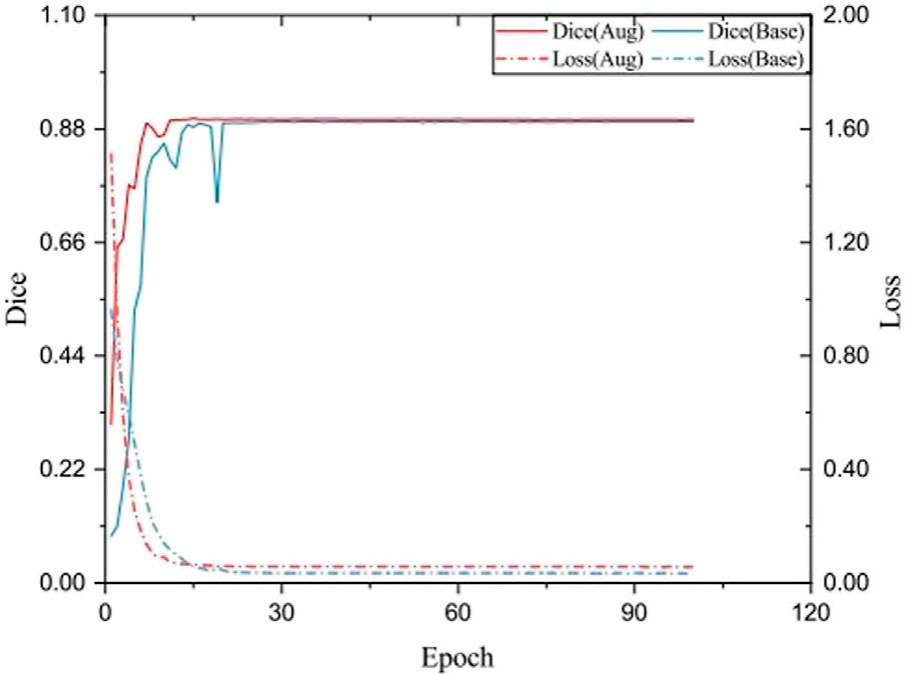
Testing Dice and training loss SeUneter with data augmentation and without data augmentation.

To verify the influence of data augmentation during training, the training loss and testing Dice in each epoch were plotted. As shown in Figure 6, the model using augmented data gradually smoothed its loss, which indicates that the model reached convergence. Without data augmentation, the drop of loss is lower, while the Dice index is relatively lower, indicating that the model produces over-fitting on the current dataset, while the data augmentation Cutout makes the data samples more differentiated and reduces the over-fitting of the model to some extent. Also, after adding the Cutout operation, its model can reach the smooth state more quickly.

### 3.3 Post-processing comparison

**FIGURE 7:**
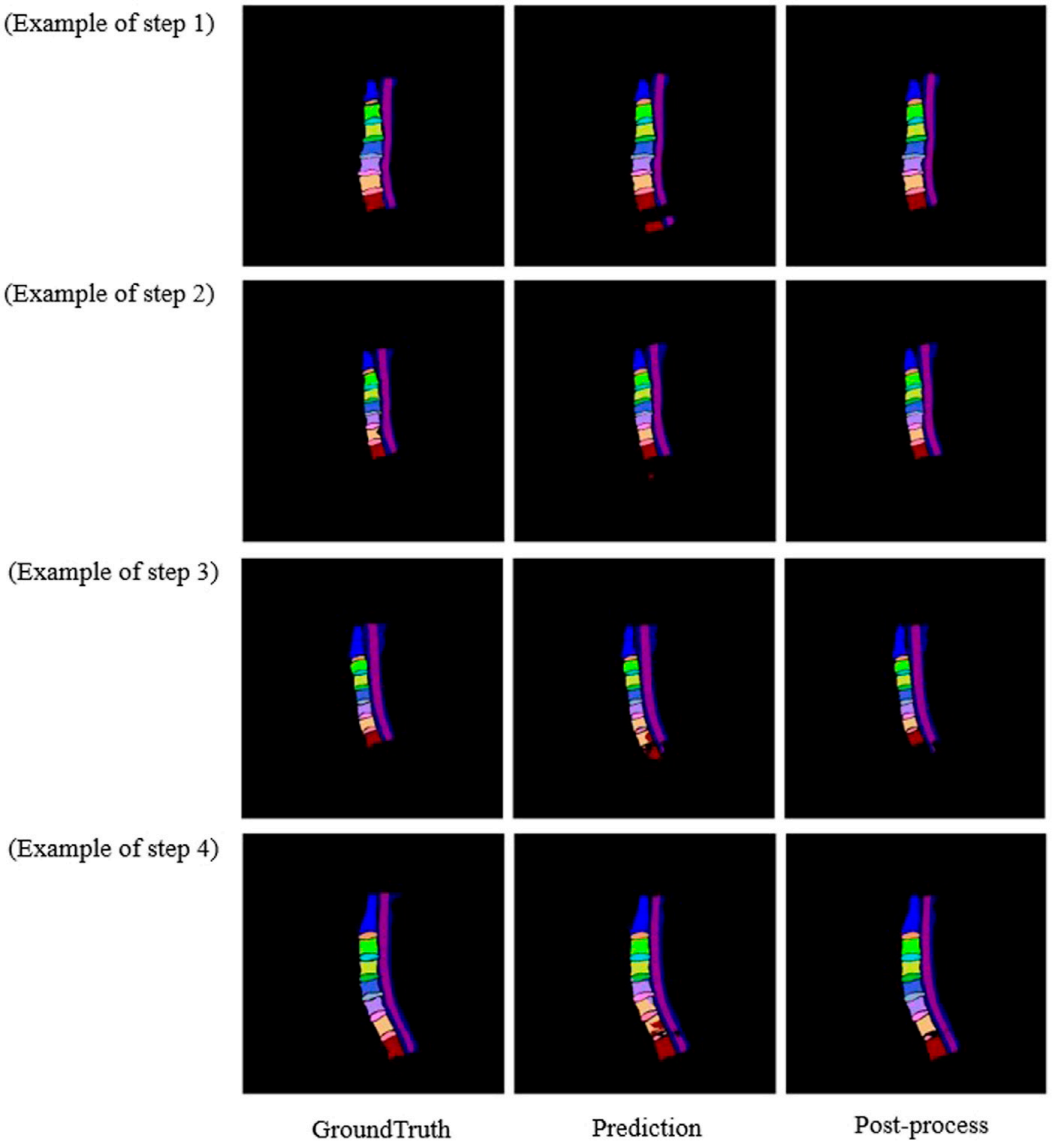
Comparison image of the original sample and the post-processing sample of the three diagrams.

To prove the effectiveness of post-processing, the results of multiple processes of post-processing are verified according to different results. As showed in Table 2, after adopting post-processing, the mDSC was improved (90.67 ± 1.63 *vs.* 90.14 ± 1.62) but did not reach a level of statistical significance (*p* = 0.506). According to Figure 7, after post-processing, abnormal segmentation was corrected and the edges were segmented more precisely, thus more closely matching the ground truth.

**TABLE 2:** Post-processing performance (%).

Metrics	Origin	Step-1	Step-2	Step-3	Step-4
DSC (±STD)	90.14 ± 1.62	90.16 ± 1.57	90.16 ± 2.18	90.67 ± 1.33	90.67 ± 1.63

Note: DSC, Dice similarity coefficient; STD, standard deviation.

### 3.4 Experimental results of multiple models

We tested the performance metrics of some strong image segmentation algorithms to verify the possible results achieved by different models. As shown in Table 3, during the measurement, the proposed SeUneter performed superior to U-Net, AttU-Net, UNet++, DeepLab-v3+, TransUNet, and Swin-Unet on the spinal cord with mIOU of 86.34% and the spinal canal with mIOU of 73.44%. The SeUneter matched or exceeded the performance of the aforementioned segmentation models when segmenting vertebral bodies or intervertebral discs.

**TABLE 3:** Mean of the IOU for a segmented class (%).

Class	Model						
	U-Net	AttU-Net	UNet++	DeepLab-v3+	TransUNet	Swin-Unet	Ours
Background	99.13	99.13	99.11	99.07	99.11	98.94	**99.21**
C2	81.89	81.01	80.69	78.29	80.5	76.9	**82.63**
C2/3	77.47	77.12	75.64	74.76	73.09	69.93	**78.21**
C3	87.17	87.61	85.54	84.15	84.42	81.37	**88.35**
C3/4	73.57	77.96	76.14	76.87	74.47	69.87	**78.95**
C4	77.39	87.48	83.79	85.24	84.44	79.50	**88.84**
C4/5	68.92	78.24	71.81	77.61	75.15	69.30	**78.84**
C5	75.22	83.81	79.11	83.57	82.89	77.03	**85.44**
C5/6	67.65	75.87	69.96	75.54	73.52	68.80	**76.47**
C6	76.8	81.39	78.47	84.03	83.3	76.31	**85.31**
C6/7	69.47	70.74	69.14	**77.13**	74.85	67.52	76.13
C7	76.17	77.37	74.50	**85.79**	85.14	77.68	84.97
C7/Tl	70.46	70.41	63.99	**78.29**	74.61	64.70	76.60
T1	77.35	77.53	73.68	**85.5**	85.9	75.07	83.93
Spinal cord	85.46	85.55	85.49	84.86	83.84	79.95	**86.34**
Spinal canal	72.47	72.82	72.43	70.59	66.89	62.94	**73.44**

Note: IOU, Intersection Over Union.

Bold represents the largest value of the row.

In addition, Table 4 showed that our proposed SeUneter achieved the highest mIOU of 82.73%, outperforming U-Net (82.73 ± 1.59 *vs.* 77.29 ± 1.45; *p* < 0.001), AttU-Net (82.73 ± 1.59 *vs.* 80.25 ± 1.55; *p* = 0.002), UNet++ (82.73 ± 1.59 *vs.* 77.47 ± 1.58; *p* < 0.001), TransUNet (82.73 ± 1.59 *vs.* 80.13 ± 1.64; *p* = 0.001), and Swin-Unet (82.73 ± 1.59 *vs.* 74.74 ± 2.06, *p* < 0.001). The mDSC of SeUneter was higher than that of DeepLab-v3+ (82.73 ± 1.59 *vs.* 81.33 ± 1.54) but did not reach a level of statistical significance (*p* = 0.073).

**TABLE 4:** Metrics for each model (%).

Metrics	U-Net	AttU-Net	UNet++	DeepLab-v3+	TransUNet	Swin-Unet	Ours (U-Net + A1 + A2 + A3 + A4)
IOU (±STD)	77.29 ± 1.45	80.25 ± 1.55	77.47 ± 1.58	81.33 ± 1.54	80.13 ± 1.64	74.74 ± 2.06	82.73 ± 1.59
DSC (±STD)	85.09 ± 1.65	87.68 ± 1.55	85.08 ± 1.62	88.78 ± 1.78	87.9 ± 1.53	84.51 ± 1.55	90.67 ± 1.31

Note: IOU, Intersection Over Union; DSC, Dice similarity coefficient; STD, standard deviation. A1 means adding the SeNet module, A2 increases the model depth in the previous process, A3 adds data enhancement operations, and A4 introduces post-processing operations in the segmentation results to optimize the segmentation performance.

Furthermore, our proposed SeUneter achieved the highest mDSC of 90.67%, outperforming U-Net (90.67 ± 1.31 *vs.* 85.09 ± 1.65; *p* < 0.001), AttU-Net (90.67 ± 1.31 *vs.* 87.68 ± 1.55; *p* < 0.001), UNet++ (90.67 ± 1.31 *vs.* 85.08 ± 1.62; *p* < 0.001), DeepLab-v3+ (90.67 ± 1.31 *vs.* 88.78 ± 1.78; *p* = 0.016), TransUNet (90.67 ± 1.31 *vs.* 87.9 ± 1.53; *p* = 0.001), and Swin-Unet (90.67 ± 1.31 *vs.* 84.51 ± 1.55; *p* < 0.001).

**FIGURE 8:**
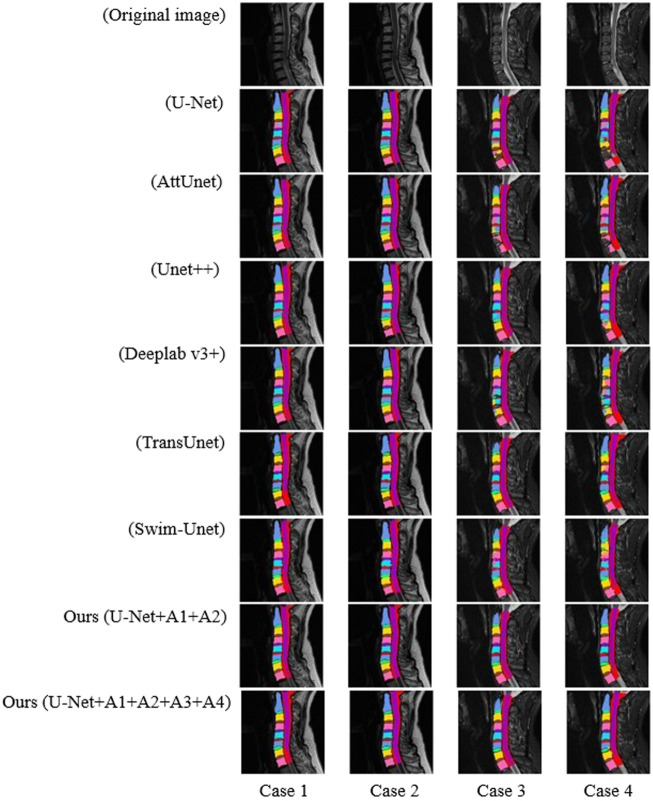
Segmentation effect on different models for two patients with a total of four legends.

As shown in Figure 8, the SeUneter model could segment the model more clearly in terms of pixel points, and the prediction of the edges was smoother compared with other networks.

## 4 Conclusion

In this paper, we collect and label a new medical image segmentation dataset for cervical MRI. We propose an improved method called SeUneter, which is based on the U-Net network by deepening the network structure and introducing channel attention to adapt the feature information of the current dataset. Furthermore, the proposed method using the characteristics of the data to construct its prior knowledge and correct the deficiencies of the model prediction performance achieves slightly better results than the current high-level segmentation methods in the cervical spine segmentation task. In future works, further exploration of model over-fitting can be attempted to improve the robustness of the current dataset in different models. Future works can be further enriched. The transformer may be further used to compensate for the lack of the CNN convolutional feature extraction and build multi-scale feature fusions to enhance the information of the sample itself to further optimize the segmentation performance of the model.

## Data Availability

The original contributions presented in the study are included in the article/Supplementary Material; further inquiries can be directed to the corresponding authors.
